# Educational paper: Connective tissue disorders with vascular involvement: from gene to therapy

**DOI:** 10.1007/s00431-012-1773-x

**Published:** 2012-07-17

**Authors:** Lut Van Laer, Dorien Proost, Bart L. Loeys

**Affiliations:** 1 Center for Medical Genetics, Faculty of Medicine and Health Sciences, University of Antwerp and Antwerp University Hospital, Prins Boudewijnlaan 43, 2650 Antwerp, Edegem Belgium; 2 Department of Human Genetics, Nijmegen Centre for Molecular Life Sciences and Institute for Genetic and Metabolic Disorders, Radboud University Nijmegen Medical Centre, Nijmegen, The Netherlands

**Keywords:** Heritable connective tissue disease, Thoracic aortic aneurysm, Marfan syndrome, Loeys-Dietz syndrome, TGFβ signaling

## Abstract

Heritable connective tissue diseases comprise a heterogeneous group of multisystemic disorders that are characterized by significant morbidity and mortality. These disorders do not merely result from defects in the amount or structure of one of the components of the extracellular matrix, as the extracellular matrix also serves other functions, including sequestration of cytokines, such as transforming growth factor beta (TGFβ). Indeed, disturbed TGFβ signaling was demonstrated in several heritable connective tissue diseases, including syndromic forms such as Marfan or Loeys-Dietz syndrome and non-syndromic presentations of thoracic aortic aneurysm/dissection. Because of these findings, new therapeutic targets have been unveiled, leading to the initiation of large clinical trials with angiotensin II type 1 receptor antagonists that also have an inhibiting effect on TGFβ signaling. Here, we present an overview of the clinical characteristics, the molecular findings, and the therapeutic strategies for the currently known syndromic and non-syndromic forms of thoracic aortic aneurysm/dissection.

## Introduction

The extracellular matrix (ECM) is a highly organized multimolecular structure that is indispensable for the normal functioning of organ systems. Heritable connective tissue diseases (HCTD) comprise a heterogeneous group of multisystemic disorders that result from genetic defects affecting normal ECM assembly and maintenance. HCTD are characterized by significant morbidity and mortality [43]. Up to now, most genes shown to be implicated in HCTD encode structural connective tissue proteins, such as collagens, fibrillin and fibronectin, or enzymes involved in the biosynthesis or processing of those proteins. It has been generally assumed that defects in the amount or structure of one of these ECM components affect normal organization and structural integrity of the supporting connective tissues and result in the typical weakness of bones, skin, vascular or other tissues, which characterizes the individual disease phenotypes. During the past decade, however, this viewpoint was found to be an oversimplification. This became apparent when other functions were discovered for the ECM, exemplified by the sequestration function for TGFβ [36], and the identification of various non-structural genes for HCTD [5, 22, 26, 29]. This implies that the pathogenesis of HCTD and the organization of the ECM are far more complex than originally thought. Thus, the current perception of the ECM is that of a complex network that, besides its mechanical role in providing strength and support to the tissues, also acts as an important reservoir for cytokines and growth factors implicated in cellular proliferation, differentiation, migration, and survival and thus has an important regulatory function in the development and homeostasis of body organs and tissues [43]. Interestingly, this fresh view on ECM function has provided new means of therapeutic intervention. Originally, when ECM was assigned a merely structural role, the only putative therapy was replacement of the deficient ECM component by gene therapy, something that seemed unachievable. Nowadays, however, with the recognition of a regulatory function for ECM components, intervening in the relevant regulatory pathways using (existing) pharmaceuticals may provide a plausible therapeutic approach in case of deficiency of an ECM component. As such, some HCTD, including Marfan (MFS) and Loeys-Dietz (LDS) syndromes, and related disorders have evolved from “incurable” genetic disorders (apart from surgical interventions) towards diseases in which hopes for new therapies have risen. Therefore, we will focus this review on these HCTD and leave other HCTDs including the Ehlers-Danlos syndromes (EDS, with the exception of vascular EDS), skeletal dysplasias, osteogenesis imperfecta, pseudoxanthoma elasticum and others largely untouched. For these HCTD, we would like to refer to other recent reviews [9, 13, 33, 49].

## Marfan syndrome

The Marfan syndrome (MFS; MIM#154700) is an autosomal dominant HCTD that occurs in approximately 1/5,000 individuals. It is a multisystemic disorder, mainly affecting the skeleton (long bone overgrowth, pectus deformity, and arachnodactyly), the eyes (lens dislocation), and the cardiovascular system. Cardiovascular pathology includes aortic root dilatation primarily at the level of the sinuses of Valsalva, dissection, and rupture, and is the leading cause of mortality [46]. Other cardiovascular manifestations include mitral valve prolapse, mitral regurgitation, (supra)ventricular arrhythmias, and systolic and diastolic left ventricular functional impairment. Each of these cardiovascular complications may present as early as infancy or may not be diagnosed until adulthood [54]. Other extra-cardiovascular manifestations include dural ectasia, *striae distensae*, recurrent inguinal hernias, pneumothorax, and lung emphysema.

In 1991, *FBN1* (encoding fibrillin-1, a structural component of the microfibrils) was identified as the gene responsible for MFS [11]. Up to now, several hundred *FBN1* mutations have been identified throughout the entire gene, but no major correlation between the nature of the mutation and the clinical phenotype has emerged [27]. Moreover, despite the presence of identical *FBN1* mutations, a remarkable intrafamilial variability is observed, in addition to a marked interfamilial variability, which has led to the hypothesis that modifier genes must be involved.

Until recently, the clinical diagnosis was based on the Ghent nosology [8], but since its revision in 2010 [30], clinical geneticists have adopted the new version [12, 40, 44]. The revised Ghent nosology puts more weight on cardiovascular manifestations, ectopia lentis, and molecular *FBN1* testing and summarizes the diagnostic decision making in a few simple rules depending on the absence or the presence of a family history [30].

Originally, it was assumed that the pathophysiology of MFS was due to structural deficiency of fibrillin-1, leading to weakened microfibrils. This hypothesis provided a plausible explanation for the vascular pathology and the lens dislocation, but other features such as skeletal overgrowth and thickening of the cardiac valves remained unexplained. Subsequently, the study of MFS mouse models suggested an important role for the cytokine transforming growth factor beta (TGFβ) in the pathogenesis of MFS; *Fbn1* deficient mice showed increased TGFβ activation and signaling and TGFβ antagonism rescued various MFS phenotypes [19, 36, 37]. The current pathogenetic model for MFS therefore suggests that the fibrillin-1 deficient state leads to dysregulation of the TGFβ signaling cascade. Although historically, studies have focused on canonical TGFβ signaling, growing evidence now shows that non-canonical signaling pathways such as those involving the MAPKs (mitogen-activated protein kinases), including the extracellular signal-regulated kinase (ERK1/2) and the Jun N-terminal kinase (JNK) also have an important role in aneurysm development [20].

## Loeys-Dietz syndrome

In 2005, a novel autosomal dominant HCTD with widespread systemic involvement was described by Loeys and Dietz. The Loeys-Dietz syndrome (LDS; MIM#609192) is typically characterized by the triad of hypertelorism (widely spaced eyes), bifid uvula/cleft palate, and arterial tortuosity with aortic aneurysm and dissection [29]. Additional manifestations include craniosynostosis, Chiari malformation, club feet, patent ductus arteriosus, and aneurysms/dissection throughout the arterial tree. LDS type 1 patients present with typical craniofacial features (craniosynostosis, cleft palate, or hypertelorism), while LDS type 2 patients mostly lack the craniofacial features but present with cutaneous findings (velvety and translucent skin, easy bruising, and atrophic scars). Despite some clinical overlap with MFS, including aortic aneurysm, arachnodactyly, pectus deformity, dural ectasia, and scoliosis, LDS patients do not present with significant long bone overgrowth or lens dislocation. Aneurysm development in LDS is more aggressive compared to that observed in MFS, i.e., on average, dissection and rupture occur at younger ages and at smaller aortic diameters than in MFS patients leading to a mean age at death of 26.0 years [32]. This implies that earlier surgical intervention (at smaller aortic diameters than in MFS patients) is indicated. Clinicians should also be aware of the increased risk of pregnancy-related complications. So far, no set of minimal diagnostic criteria have been established for LDS and the diagnosis should thus be confirmed through molecular genetic testing.

LDS is caused by mutations in genes encoding transforming growth factor-beta receptor 1 or 2 (*TGFBR1* or *TGFBR2*) [29]. No clinical distinction can be made between *TGFBR1* or *TGFBR2* mutation carriers. Comparable to MFS, a remarkable inter-individual and intrafamilial clinical variability is observed. The mutations mainly affect the serine-threonine kinase domain of both receptors and lead to loss-of-function. Interestingly and paradoxically, TGFβ signaling was enhanced in aortic walls and fibroblast cultures of LDS patients [29]. After the finding of increased TGFβ signaling in a MFS mouse model [36], the discovery that mutations in *TGFBR1/2* were the cause of another aneurysmal syndrome and the fact that these loss-of-function mutations led to a paradoxical activation of TGFβ signaling provided further proof for an important role of TGFβ signaling in aneurysma development [29].

## Other disorders in the MFS/LDS spectrum

### The aneurysm-osteoarthritis syndrome or Loeys-Dietz syndrome, type 1C

Van de Laar et al*.* [51] recently described another HCTD, designated aneurysms-osteoarthritis syndrome (AOS) or Loeys-Dietz syndrome, type 1C (MIM#613795). AOS is characterized by aneurysms, dissections, and tortuosity throughout the arterial tree in addition to craniofacial (including hypertelorism and abnormal palate/uvula), skeletal (including arachnodactyly and scoliosis), and cutaneous (including striae and velvety skin) features and thus perfectly fits in the phenotypic spectrum of LDS. A distinguishing feature, however, is the presence of early-onset osteoarthritis. AOS is caused by mutations in the gene encoding SMAD3, a component of the canonical TGFβ signaling pathway [51–53]. Again, dysregulation of TGFβ signaling was demonstrated when studying aortic walls from AOS patients. All investigated markers of TGFβ signaling, including pSMAD2, SMAD3, and CTGF (connective tissue growth factor) were significantly increased in these tissues [51].

### Vascular Ehlers-Danlos syndrome

EDS comprises a clinically and genetically diverse group of HCTD, characterized by congenital fragility of the connective tissues. The Villefranche nosology recognizes six subtypes based on clinical characteristics, inheritance pattern, biochemical, and molecular findings [1]. The most common types of EDS are the classic, the hypermobile and the vascular type, while the kyphoscoliotic, the arthrochalasis, and the dermatosparaxis type are rather rare. Here, we restrict ourselves to the vascular type of EDS (MIM#130050) since the latter is associated with a high risk for life threatening complications and as a consequence with a decreased life expectancy. We refer to a recent review for details on the other EDS types [9].

Typical clinical manifestations of vascular EDS include thin, translucent skin, characteristic facial appearance, vascular fragility demonstrated by extensive bruising and easy bleeding and spontaneous arterial/intestinal/uterine ruptures [43]. Vascular EDS is caused by mutations in *COL3A1* (type III collagen α-chain 1) [47]. These mutations consist mostly of missense mutations that lead to the substitution of essential glycine residues within the triple helical domain of the type III collagen chain. Interestingly, in a cohort of 40 patients displaying a vascular EDS-like phenotype but normal collagen III biochemistry, 30 % carried *TGFBR1*/2 mutations [32], suggesting on the one hand that vascular EDS closely resembles LDS but on the other hand that *TGFBR* mutations may cause a broad spectrum of diseases associated with aortic aneurysms. To the best of our knowledge, no demonstration of dysregulated TGFβ signaling in vascular EDS has been published so far.

### Arterial tortuosity syndrome

The arterial tortuosity syndrome (ATS; MIM#208050) is an autosomal recessive HCTD that is characterized by tortuosity, elongation, stenosis, and aneurysm formation in the major arteries. Patients often die at a young age. Features in common with LDS include arachnodactyly, hypertelorism, cleft palate and/or bifid uvula, joint laxity or contractions, and micro/retrognathia. ATS is caused by loss-of-function mutations in *SLC2A10*, encoding GLUT10, which belongs to the glucose transporter family. ATS has been associated with enhanced TGFβ signaling, as demonstrated by increased pSMAD2 and CTGF immunostaining in patients' arterial walls [5].

## Cutis laxa

Hereditary cutis laxa (CL) delineates a heterogeneous group of rare HCTD, characterized by the presence of loose, sagging, inelastic skin in addition to systemic manifestations of variable severity [43]. Both autosomal dominant (ADCL) and autosomal recessive forms (ARCL) exist. ADCL is relatively benign compared to ARCL. In ADCL, the typical loose, sagging skin can be accompanied by gastrointestinal diverticulae, hernias, and genital prolapse. Also, pulmonary artery stenosis, aortic aneurysm, bronchiectasis, and emphysema may occur. The major causal gene for ADCL is the elastin gene (ELN; MIM #123700), while one tandem duplication in the fibulin-5 gene (*FBLN5*; MIM#614434) has been described as well. Gain-of-function mutations in ELN lead to ADCL, whereas loss-of-function point mutations or contiguous gene deletions (involving ELN) give rise to isolated supravalvular aortic stenosis and Williams-Beuren syndrome, respectively.

ARCL type 1 (ARCL1) is a life-threatening disorder characterized by vascular anomalies, lung emphysema, and diverticulae of the urinary and gastrointestinal tract aside from the cutaneous manifestations. The prognosis of ARCL1 is poor as cardiopulmonary failure severely limits the lifespan of these patients. Mutations in two fibulin genes, *FBLN4* (MIM#614437; also designated *EFEMP2)* or *FBLN5* (MIM#219100), are responsible for ARCL1 [21, 28]. Vascular involvement (arterial aneurysms, arterial tortuosity), however, is most probably restricted to *FBLN4* mutations [6, 42], while the cutaneous manifestations in *FBLN4* patients are limited and are more pronounced in *FBLN5* patients. As such, ARCL1 caused by *FBLN4* mutations can be categorized within the LDS spectrum.

Another autosomal recessive syndrome, closely resembling ARCL1 and designated Urban-Rifkin-Davies syndrome (URDS; MIM#613177), was recently shown to be caused by mutations in *LTBP4* [50]. *LTBP4* patients present with severe gastro-intestinal and urinary tract involvement in addition to loose, sagging skin. Aside from peripheral pulmonary artery stenosis, *LTBP4* patients have no vascular involvement. The gene product, LTBP4, belongs to the latent transforming growth factor beta binding proteins (LTBPs), which associate with TGFβ when secreted into the ECM and keep TGFβ in its latent form. The latent complexes play an important role in the regulation of TGFβ-mediated signaling.

Interestingly, both in ADCL caused by *ELN* mutations, ARCL1 caused by *FBLN4* mutations and in URDS, dysregulation of TGF-beta signaling was observed [4, 42, 50].

Other types of cutis laxa and related diseases exist (ARCL2, ARCL3, wrinkly skin syndrome, gerodermia osteodysplastica, etc.). However, as so far no involvement of TGFβ signaling has been suggested for these conditions, we prefer not to go into detail and to refer to a recent review by Berk et al*.* [2] for a more thorough description of these HCTD.

## Non-syndromic thoracic aortic aneurysm and dissection

Non-syndromic types of thoracic aortic aneurysms and dissections (TAAD) or types in which only minor additional features are present exist as well. Occasionally, mutations in *FBN1* [34] and in *TGFBR1/2* [48] causing TAAD have been described, perhaps representing the mildest end of the MFS/LDS phenotypic spectrum.

In addition, *ACTA2* mutations have been identified in 14 % of TAAD patients [16], while *MYH11* mutations have been found in TAAD patients with persistent ductus arteriosus [56]. Additional symptoms that can be found in *ACTA2* mutation positive patients include persistent ductus arteriosus, bicuspid aortic valve, iris flocculi, and cerebrovascular accidents. In fact, *ACTA2* mutations can also cause stroke, Moya-Moya disease, and coronary artery disease [17]. *ACTA2* and *MYH11* encode the smooth muscle cell specific α-actin and β-myosin heavy chain, respectively. Both proteins are indispensable components of the smooth muscle cells contractile apparatus and mutations in the encoding genes may prevent proper contraction of the smooth muscle cells. Interestingly, enhanced TGFβ signaling was demonstrated in aortic tissue derived from both *ACTA2* and *MYH11* patients [41]. Additionally, Gomez et al. [15] found a dysregulation of TGFβ signaling in ascending aortic walls of non-syndromic thoracic aneurysm patients.

## Recent new findings

From the above, a rule seems to have emerged: TGFβ signaling is significantly associated with both syndromic and non-syndromic types of thoracic aortic aneurysms. More importantly, as TGFβ antagonism can attenuate or prevent the various phenotypes, a causal relationship seems highly plausible. However, the URDS, one of the disorders mentioned above, is an exception to this rule, i.e., enhanced TGFβ signaling was found, but URDS patients do not present with thoracic aortic aneurysms. Here, a few additional examples are elaborated to illustrate that increased TGFβ signaling does not always lead to thoracic aortic aneurysm development and that the overall picture is thus far more complex.

In the stiff skin syndrome (SSS; MIM#184900), a rare, autosomal dominant condition of congenital scleroderma (thickened skin) associated with short stature, domain-specific *FBN1* mutations have been identified. All mutations were positioned in two *FBN1* exons that encode the fourth TGFβ binding protein-like domain (TB4). This domain contains the RGD (arginine–glycine–aspartic acid) motif, which mediates integrin binding. SSS patients do not show the typical MFS symptoms, including long bone overgrowth, thoracic aortic aneurysms, ectopia lentis and joint laxity; nor do MFS patients ever present with skin fibrosis. Still, increased TGFβ concentration and signaling was observed in the dermis of SSS patients [31]. Given the restricted nature of the mutations and limited affected organs observed in SSS (mainly skin), it was hypothesized that SSS mutations may lead to a gain-of-microfibrillar-function, while most MFS mutations clearly result in loss of function [31].

Recently, mutations restricted to two exons encoding the TB5 domain of *FBN1* were shown to lead to acromicric (AD; MIM#102370) and autosomal dominant geleophysic dysplasia (GD; MIM#614185) [24]. Both syndromes are characterized by severe short stature, short extremities, and stiff joints. Thoracic aortic aneurysms are not part of the phenotypic spectrum, although cardiac valves stenosis and insufficiency have been associated with GD. As such, the AD and GD spectrum again opposes the MFS spectrum. Still, enhanced TGFβ signaling was demonstrated irrefutably in fibroblasts of these patients. As AD had been shown previously to be caused by mutations in *ADAMTSL2* [25] and a direct interaction between FBN1 and ADAMTSL2 was found, the authors suggested that a dysregulation of the FBN1/ADAMTSL2/TGFβ interrelationship lay at the basis of the AD/GD phenotypes.

These few examples further illustrate the complexity of the TGFβ signaling pathways and their regulatory levels. Most likely, several factors, including the nature and the location of the mutation, the nature of the affected gene product, its spatiotemporal expression levels and the influence of other paracrine or autocrine factors influence the final outcome. Additional research will thus be necessary to understand all contributing factors in order to be able to predict aneurysm formation.

## Treatment strategies

Traditionally, management of MFS patients consisted of a regular evaluation of their aortic diameters and therapeutic treatment with beta-blockers [45]. For MFS patients, surgical intervention is indicated when the aortic diameter exceeds 5 cm or when the aneurysmal growth exceeds 1 cm/year. For LDS, surgical intervention at smaller aortic diameters is recommended. In addition, as the aneurysms in LDS patients can be found throughout the arterial tree, more extensive imaging is mandatory.

Interestingly, the identification of enhanced TGFβ signaling in MFS mouse models has led to the identification of losartan as an exciting new possible therapy for MFS [18, 19], as losartan also has an inhibiting effect on TGFβ signaling besides being an antagonist of the angiotensin II type 1 receptor. Indeed, losartan outperformed the beta-blocker atenolol when applied to a MFS mouse model [19]. This exiting finding led to a preliminary observational study in seventeen pediatric MFS patients in whom other therapy had failed to prevent progressive aortic root dilatation [3]. These patients were treated with losartan during 12 to 47 months, leading to a reduction in rate of aortic root diameter growth from 3.54 ± 2.87 mm/year to 0.46 ± 0.62 mm/year. As this preliminary study had provided proof that losartan can be efficient in human patients as well, several large, randomized clinical trials on MFS patients have been initiated since. A first trial is comparing atenolol with losartan in approximately 600 MFS patients during a 3-year period. Unless the trial is stopped early for treatment benefit, results can be expected by 2014 [23]. Additional trials with various designs and inclusion criteria are currently ongoing in Belgium, France, Italy, The Netherlands, Taiwan, and the United Kingdom [10, 14, 35, 39]. The next few years will thus reveal whether losartan will become the preferred therapeutic in MFS patients.

Because of the less frequent nature of related disorders with aneurysm formation and TGFβ signaling upregulation (LDS, AOS, ATS, ADCL, ARCL1, TAAD), it is unlikely that large, randomized trials can be organized for these conditions. For these rarer conditions, evidence may be gathered through the study of relevant animal models, after which cautious treatment of patients may be considered. Prudence is indeed called for, because in contrast to the findings in thoracic aortic aneurysms, it was shown in a validated mouse model of abdominal aortic aneurysm formation (Ang II-induced; [7]), that TGFβ activity actually protects against aortic aneurysm progression and that TGFβ neutralization promotes aortic aneurysm formation [55].

Although no causal treatment exists for EDS, prophylactic measures are essential, in particular for the vascular type of EDS because of its serious nature. Individuals with vascular EDS should avoid contact sports and isometric exertion (like weightlifting). Usage of anti-coagulentia and other agents that interfere with platelet function should also be avoided. Likewise, invasive procedures, including angiography, catherizations and surgical interventions are contra-indicated. Recently, it was demonstrated that the beta-blocker celiprolol could prevent major complications in patients with vascular EDS [38], giving new hope for treatment of this devastating condition.

**Fig. 1:**
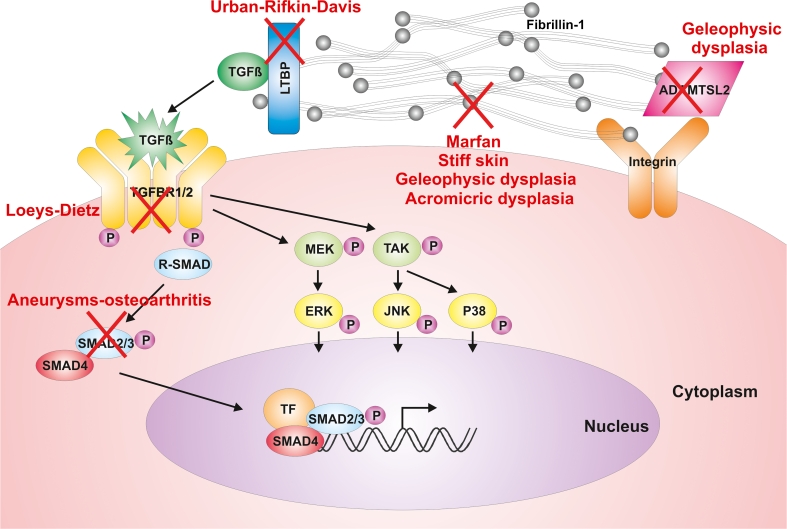
Defects within components of the ECM and the TGFβ signaling pathway leading to various HCTD. The defective components are indicated with a *red cross* and the respective diseases are listed in the vicinity of the relevant cross

Much of the information that we have reviewed here, is also summarized in Table 1 (clinical synopsis) and Fig. 1 (defects within components of the ECM and the TGFβ signaling pathway).

**Table 1:** Synopsis of the clinical features

Disorder/syndrome	Gene	Cardiovascular			Skeletal	Eye	Cutaneous	Cranio-facial
		Aneurysm	Tortuosity	Other				
Marfan	FBN1	+++	−	MVP	+++	+++	+	+
Loeys-Dietz	TGFBR1/2	+++	++	BAV, PDA	++	++	++	++
Aneurysms-osteoarthritis	SMAD3	+++	++	BAV, PDA	++	+	++	++
Vascular Ehlers-Danlos	COL3A1	++^a^	−		+	−	++	++
Arterial tortuosity syndrome	SLC2A10	+	+++	PS	+	++	+	++
Autosomal dominant cutis laxa	ELN	+	−		−	−	+++	++
Autosomal recessive cutis laxa type 1	FBLN4	++	++		+	−	++	++
Autosomal recessive cutis laxa type 1	FBLN5	−	−	SVAS, PS	−	−	+++	+
Urban-Rifkin-Davis	LTBP4	−	−	PS	−	−	+++	++
Thoracic aortic aneurysm/dissection	ACTA2	+++	−	BAV, PDA	−	+^c^	+^d^	−
Thoracic aortic aneurysm/dissection	MYH11	+++	−	PDA	−	−	−	−
Stiff skin syndrome	FBN1	−	−	−	++^b^	−	+++	−
Acromicric/geleophysic dysplasia	FBN1	−	−	AS	++^b^	−	++	++
Geleophysic dysplasia	ADAMTSL2	−	−	AS	++^b^	−	++	++

^a^Vascular fragility

^b^Short stature instead of long stature

^c^Iris flocculi

^d^Livedo reticularis

*MVP* mitral valve prolapse; *BAV* bicuspid aortic valve; *PDA* patent ductus arteriosus; *PS* pulmonary artery stenosis; *SVAS* supravalvular aortic stenosis; *AS* aortic stenosis
